# The 23^rd^ Annual Meeting of the European Tissue Repair Society (ETRS) in Reims, France

**DOI:** 10.1186/1755-1536-7-3

**Published:** 2014-02-19

**Authors:** Johannes W Von den Hoff, Magnus S Ågren, Bernard Coulomb, Sabine A Eming, Jean-Jacques Lataillade

**Affiliations:** 1 Department of Orthodontics and Craniofacial Biology, Radboud University Medical Center, Nijmegen, The Netherlands; 2 Department of Clinical Medicine, Faculty of Health and Medical Sciences, and Copenhagen Wound Healing Center and Digestive Disease Center, Bispebjerg Hospital, University of Copenhagen, Copenhagen, Denmark; 3 Inserm, U970, PARCC/HEGP, Paris, France; 4 Department of Dermatology, University of Cologne, Köln, Germany; 5 Department of Research and Cell Therapy, Percy Military Hospital, Clamart, France

**Keywords:** Tissue repair, Wound healing, Regenerative medicine, Stem cells, Biomaterials, Infection, Burns

## Abstract

The 23^rd^ Annual Meeting of the European Tissue Repair Society, Reims, France, October 23 to 25, 2013 focused on tissue repair and regenerative medicine covering topics such as stem cells, biomaterials, tissue engineering, and burns.

## Introduction

**Figure 1:**
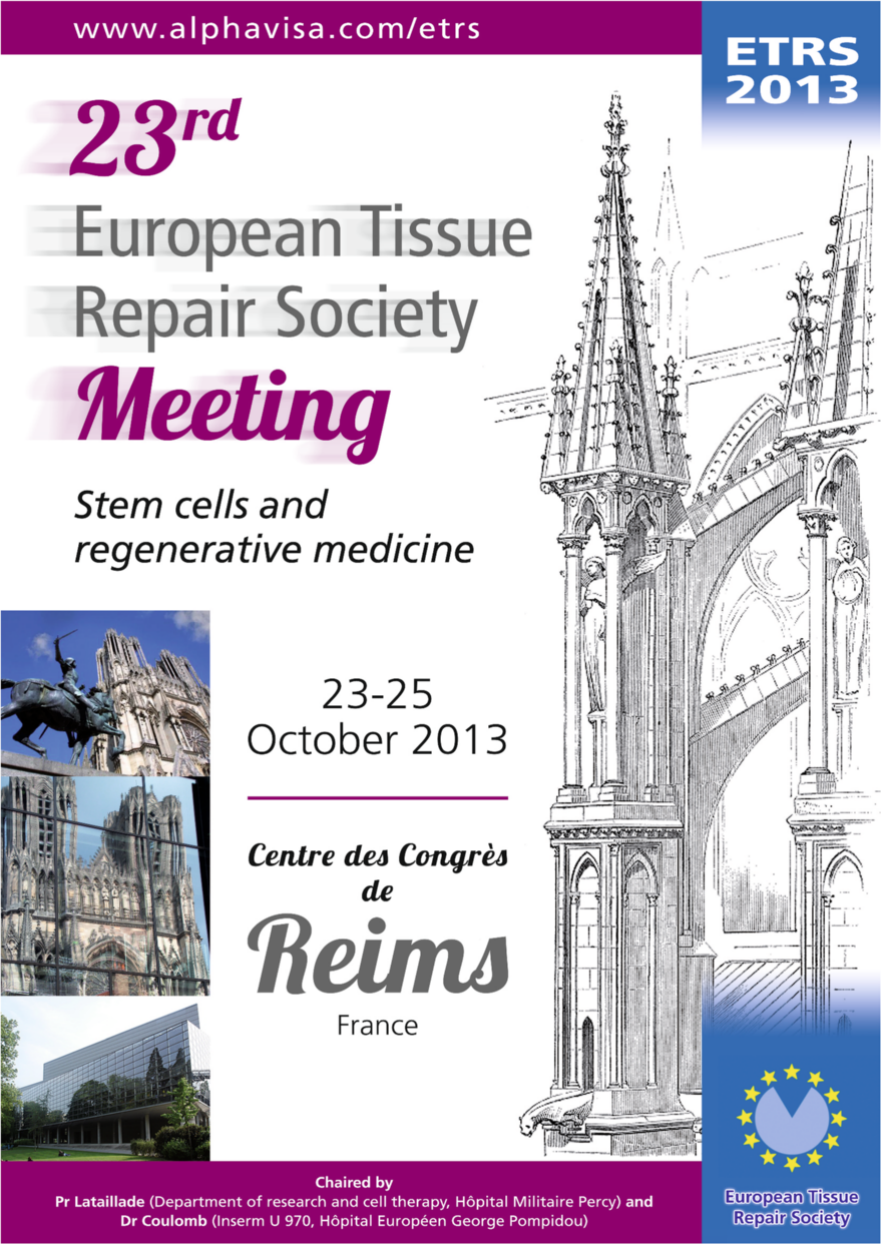
**Flyer of the 23**^**rd **^**Annual Meeting of the European Tissue Repair Society.**

The 23^rd^ Annual Meeting of the European Tissue Repair Society (ETRS) was organized by Jean-Jacques Lataillade and Bernard Coulomb in the Reims Congress Center, Reims, France from 23 to 25 October 2013 (Figure 1). The 189 attendees included clinical, industrial, and basic scientists, of which 54 were students. Eighteen countries were represented, several from outside of Europe (USA, Australia, Canada, Japan, South Korea, and Mexico).

The general theme “Stem cells and regenerative medicine”, continued the tradition of previous meetings to join with local societies. Seven of the 10 sessions were organized in association with French research and clinical societies: “Scar and reconstructive surgery” (Société Française et Francophone des Plaies et Cicatrisation), “Cardiovascular repair”, “Matrix and Biofilm” (Société Française de la Biologie de la Matrice ExtraCellulaire), “Burns” (Société Française de Traitement et d’Etude de la Brûlure), “Biomaterials” (Association pour le développement des Biomatériaux), “Stem cell niche and rejuvenation”, “Cell and tissue engineering” (Société Française de Bio-ingénierie Cellulaire et Tissulaire). The European Wound Management Association guest session was dedicated to infection.

The French Societies also contributed to the 10 awards for Young Investigators, this year adding up to 3.500€. The ETRS award for the best oral presentation, which includes an invitation to the 2014 Annual Meeting of the Wound Healing Society (WHS) in Orlando, Florida, was won by Raya Bushkalova (Inserm, Toulouse, France). Her winning presentation was entitled “Polyelectrolyte scaffolds for cardiac mesenchymal stem cell therapy”.

The interaction with the WHS was reinforced by the attendance of Paul Liu (WHS President) and Bob Diegelmann (WHS past President) together with a total of 13 attendees from the United States. The Australasian Wound & Tissue Repair Society (AWTRS) was also well-represented by 4 active members including Allison Cowin (AWTRS past President), giving the opportunity to strengthen the exchange between the societies. Interaction with industry was fostered through 5 “Industrial Forums” (Healthpoint, Laboratoires Brothier, MacoPharma, Médicen, and Terumo BCT) and lively exchanges at the exhibition stands. Finally, 75 posters were presented during the meeting. The following sections will give an overview of the scientific content of the meeting. Abstracts of the contributions have been published elsewhere [*Wound Repair Regen* 2013, **21:**A55-A88].

## Dynamics and consequences of the inflammatory response in tissue repair and regeneration

Evidence from different model organisms indicates that the immune system is of primary importance in determining the outcome of the repair response, including the extent of scarring as well as the restoration of organ structure and function. Yet, the functional relationship between repair, regeneration, and the immune response is complex and not completely understood. In this year’s Charles Lapière Memorial Lecture, Sabine Eming outlined positive and negative roles of the immune response in tissue repair. Her group contributed substantially to the molecular understanding of the consequences of an unbalanced inflammatory response at the wound site, leading to profound effects on downstream cell activities essential for repair. Recent data from murine wound models have identified monocytes/macrophages as critical regulators of distinct functions during the sequential stages of wound healing. This indicates their potential for monocyte-based therapies in tissue repair, and supports current clinical studies that investigate monocyte/macrophage activation to normalize tissue homeostasis. In future studies, it will be important to further dissect the beneficial and harmful effects of the inflammatory response during wound healing.

## The vascular system: from cardiovascular repair to endothelial cell heterogeneity

Human pluripotent stem cells hold great promise in regenerative medicine. However, their differentiation towards mature cells such as functional cardiomyocytes remains a major challenge. A novel protocol was presented, based on epicardium-derived cells and a 3-dimensional collagen scaffold that allows the differentiation of cardiac progenitor cells to functional cardiomyocytes capable to regenerate a failing myocardium. Another group presented further insight in signaling pathways, including Notch and hypoxia inducible factor that direct endothelial cell heterogeneity, which might have consequences for diseases affecting different parts of the vascular system. These findings can be used to generate vascular bed-specific endothelial cells from endothelial progenitor cells in revascularization therapies.

## Cellular senescence

Cellular senescence or progressive exhaustion of cell proliferation was described for the first time more than 50 years ago by Dr. Hayflick. Today, we differentiate between replicative and stress-induced cellular senescence. Both are caused by damage to DNA via the p53/p21^CIP1^ and p16^INK4A^/pRB pathways, which might be related to atherosclerosis, osteoarthritis, and chronic cutaneous wounds. It was also emphasized that senescence may be a tumor suppressive mechanism. Although senescence-associated β-galactosidase activity is used as a marker, it can only be applied to cryopreserved tissue. At the conference, lipofucin was presented as a potentially more versatile biomarker that can be detected in archival paraffin-embedded tissue.

In 2012, Professor Yamanaka received the Noble Prize for reprogramming adult human dermal fibroblasts into induced pluripotent stem cells (iPSCs) using a 4-factor cocktail (Oct4, Sox2, Klf4, c-Myc). Novel data discussed at the meeting showed that it is also possible to reprogram senescent cells into iPSCs using a 6-factor cocktail.

## Cell therapy

More than 500 companies are involved in the development of cell therapies worldwide. Culture systems for somatic stem cells are becoming more sophisticated in recapitulating the in vivo conditions. It was emphasized that 3-dimensional matrices in bioreactors will improve the yield of the desired cell phenotype. There is also a trend of replacing animal-derived serum components with mitogenic human platelet lysate. In addition, the inclusion of endothelial cells in the tissue constructs might increase the grafting success.

**Figure 2:**
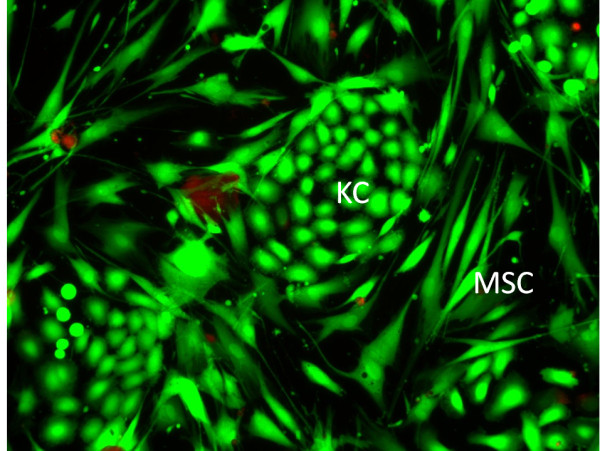
**Human keratinocyte clones within a human mesenchymal stem cell feeder layer.** KC: keratinocytes, MSC: mesenchymal stem cell.

The current status of adipose stem cell therapy in various areas of regenerative medicine was reviewed at the meeting. Interestingly, initial trials indicate that is possible to isolate adipose-derived stem cell-like cells from burn eschar. This source of stem cells may prove useful in the treatment of burn injuries. In addition, a new therapeutic strategy was presented in the use of autologous keratinocytes in treating patients with burn injuries (Figure 2).

## Developments in biomaterials and tissue engineering

Bone and cartilage are tissues that the field of tissue engineering and regenerative medicine has since long investigated. A keynote presentation focused on the communication between mesenchymal stem cells (MSCs) and endothelial progenitor cells in bone engineering, and its importance for obtaining a 3-dimensional vascularized tissue. Niche factors also determine the fate of stem cells. An interesting development is the high-throughput screening of biochemical and biophysical niche factors. For example, type I collagen microspheres impregnated with transforming growth factor-β3 and seeded with MSCs form a cartilage-like tissue in culture. Also in vivo, the microspheres induced cartilaginous tissue after subcutaneous implantation in mice. Chitosan/hydroxyapatite matrices and coral scaffolds cultured in a perfusion bioreactor might be suitable for bone engineering. Also, human amniotic membrane was shown to have osteogenic potential *in vitro* as well as *in vivo*.

Further presentations focused on the engineering of less intensively studied tissues. Recent advances in the generation of red blood cells for blood transfusion show that human embryonic stem cells and iPSCs can be used for this purpose. Gingival mucosa injuries exhibit nearly scarless healing and thereby resembling fetal tissue healing mechanisms. Gingival fibroblasts were shown to have an even higher capacity than dermal fibroblasts to promote healing in several wound models. Also in the field of oral tissue engineering, a new model for muscle regeneration in the soft palate of the rat was presented. This model may be applied in the development of new strategies to improve the healing of surgical wounds after cleft palate repair.

## Biofilm and infection

A keynote lecture explained that biofilms are communities of microorganism that are attached to a surface, with the cells encased in extracellular polymeric substance, typically composed of polysaccharides, proteins, extracellular DNA, and lipids. Biofilm is a virulence factor and especially common in severe infections that are difficult to eradicate. Aggressive surgical debridement is paramount in these cases. Negative pressure wound therapy is a useful supplement of lower limb infections. In traumatic cases, negative pressure therapy combined with an antiseptic has proven to be especially effective against osteomyelitis. Interestingly, osteomyelitis can be induced experimentally with methicillin-resistant Staphylococcus aureus (MRSA) biofilm applied to open fractures.

Pressure ulcers are often neglected but can be a risk factor for MRSA infection. Therefore, close attention should be paid to the microbiological status of these chronic wounds. The cornerstone in the treatment of infected pressure ulcers is surgical debridement. The use of antibiotics should be restricted to cases of sepsis and osteomyelitis to avoid resistance.

## Conclusion

The 23^rd^ Annual Meeting of the European Tissue Repair Society provided an exciting update of new developments in crucial areas of both fundamental and clinical aspects of wound healing and tissue repair. This meeting underlined that several stem cell protocols have already been developed into treatments in humans demonstrating that stem cell-based regenerative medicine may be translated into therapies in patients with wound healing problems. The meeting created a lively forum for interactions between professionals from different disciplines, thus reflecting the mission statement of the ETRS: “…to promote knowledge and interchange between scientists, healthcare professionals, industry and other individuals that have an interest in tissue repair of all organs”. The 24^rd^ Annual Meeting of the ETRS will take place in Edinburgh (UK) from September 11–13, 2014, and the 7^th^ Joint Meeting of the ETRS & the WHS in Copenhagen (Denmark) October 20–22, 2015.

## Abbreviations

AWTRS: Australasian Wound & Tissue Repair Society; ETRS: European Tissue Repair Society; iPSC: Induced Pluripotent Stem Cell; MRSA: Methicillin-Resistant Staphylococcus Aureus; MSC: Mesenchymal Stem Cell; WHS: Wound Healing Society.

## Competing interests

The authors declare that they have no competing interests.

## Author’s contributions

BC: Conception and design, manuscript writing and final approval of the manuscript. JJL: Conception and design, manuscript writing and final approval of the manuscript. JWV: Conception and design, manuscript writing, critical revision and final approval of the manuscript. MSÅ: Conception and design, manuscript writing and final approval of the manuscript. SAE: Conception and design, manuscript writing and final approval of the manuscript. All authors read and approved the final manuscript.

